# Presence of *Chlamydia, Mycoplasma, Ureaplasma,* and Other Bacteria in the Upper and Lower Genital Tracts of Fertile and Infertile Populations

**DOI:** 10.1155/S1064744993000201

**Published:** 1993

**Authors:** Mark G. Martens, Ronald L. Young, Marco Uribe, V. C. Buttram, Sebastian Faro

**Affiliations:** 1 Department of Obstetrics and Gynecology University of Texas Medical Branch Galveston TX 77555-0587 USA; 2 Department of Obstetrics and Gynecology Baylor College of Medicine Houston TX USA; 3 Department of Gynecology and Obstetrics University of Kansas School of Medicine Kansas City KS USA

## Abstract

*Objective:* The genital mycoplasmas (*Mycoplasma hominis* and *Ureaplasma urealyticum*) and *Chlamydia trachomatis* have been implicated as possible etiologic factors in infertility. Their role in patients with infertility needs to be further defined.

*Methods:* Seventy-nine infertile patients underwent laparoscopy with cultures obtained for aerobic and anaerobic bacteria, *Chlamydia, Mycoplasma,* and *Ureaplasma* from the peritoneal fluid, fallopian tube, endometrium, and endocervix. Cultures for similar organisms were taken from the endocervix of 80 fertile women in their first trimester. Culture results were also compared according to ovulatory status and laparoscopic findings in the infertile group.

*Results:* There were no differences in the recovery of *Ureaplasma* (29% vs. 28%) or *Chlamydia* (4% vs. 0%) positive cervical cultures in the fertile and infertile groups, respectively. However, a significantly higher number of *Mycoplasma* positive cervical cultures (14% vs. 5%, *P* =  0.05) were found in the fertile group. Only two upper genital tract cultures were found to be positive (*Ureaplasma*).

*Conclusions:* Therefore, if these organisms play a role in infertility, they are present and eradicated prior to infertility work-up and thus do not supports the use of a routine trial of antibiotics prior to laparoscopy.


Infectious Diseases in Obstetrics and Gynecology 1:85–90 (1993)
© 1993 Wiley-Liss, Inc.

### Presence of *Chlamydia, Mycoplasma, Ureaplasma,* and Other Bacteria in the Upper and Lower Genital Tracts of Fertile and Infertile Populations

**Mark G. Martens, Ronald L. Young, Marco Uribe, V.C. Buttram, Jr., and Sebastian Faro**

*Department of Obstetrics and Gynecology, University of Texas Medical Branch, Galveston (M.G.M.), and Department of Obstetrics and Gynecology, Baylor College of Medicine, Houston (R.L.Y., M.U., V.C.B.), TX; Department of Gynecology and Obstetrics, University of Kansas School of Medicine, Kansas City (S.F.), KS*

### Abstract

***Objective:*** **The genital mycoplasmas (*Mycoplasma hominis* and *Ureaplasma urealyticum*) and *Chlamydia trachomatis* have been implicated as possible etiologic factors in infertility. Their role in patients with infertility needs to be further defined.**

***Methods:*** **Seventy-nine infertile patients underwent laparoscopy with cultures obtained for aerobic and anaerobic bacteria, *Chlamydia, Mycoplasma,* and *Ureaplasma* from the peritoneal fluid, fallopian tube, endometrium, and endocervix. Cultures for similar organisms were taken from the endocervix of 80 fertile women in their first trimester. Culture results were also compared according to ovulatory status and laparoscopic findings in the infertile group.**

***Results:*** **There were no differences in the recovery of *Ureaplasma* (29% vs. 28%) or *Chlamydia* (4% vs. 0%) positive cervical cultures in the fertile and infertile groups, respectively. However, a significantly higher number of *Mycoplasma* positive cervical cultures (14% vs. 5%, $P = 0.05$) were found in the fertile group. Only two upper genital tract cultures were found to be positive (*Ureaplasma*).**

***Conclusions:*** **Therefore, if these organisms play a role in infertility, they are present and eradicated prior to infertility work-up and thus do not support the use of a routine trial of antibiotics prior to laparoscopy.** © 1993 Wiley-Liss, Inc.

Key words

*Chlamydia, Mycoplasma, Ureaplasma,* infertility

Over the past several decades, remarkable progress has been made in the treatment of infertility. There still, however, continues to be a subset of couples with reproductive failure in whom there is no demonstrable etiology. An association of the genital mycoplasmas (*Mycoplasma hominis* and *Ureaplasma urealyticum*), as well as *Chlamydia trachomatis*, with infertility has long been suspected.[1–3] However, neither epidemiologic evaluation nor treatment data from females in infertile partnerships have offered consistent reproducible results to implicate any of these organisms as etiologic agents. Since all 3 of these organisms are sensitive to currently available antibiotics, routine empiric treatment of these organisms often occurs, despite the lack of substantial proof of its efficacy.

Overall, considerable controversy exists covering the role of all 3 of these organisms in human infertility. The major problem with previous studies is that infertility is a syndrome, not a specific

**Address correspondence/reprint requests to Dr. Mark G. Martens, Department of Obstetrics and Gynecology, University of Texas Medical Branch, Galveston, TX 77555-0587.**

Received March 30, 1993

Accepted June 14, 1993

entity, and that these organisms are likely to be related only to certain clinicopathological subsets.

Much of the data conflict, and the clinician or infertility specialist is left with several questions: 1) Do the genital mycoplasmas play a pathogenic role in infertility? 2) Are cultures for *Mycoplasma* and *Ureaplasma* a necessary part of the infertility work-up? 3) Does cervical colonization correlate with upper genital tract involvement? 4) Will treatment result in increased fertility rates?

Most of the studies include patients with "idiopathic infertility," of whom many have had inadequate diagnostic evaluations. If *Mycoplasma*, *Ureaplasma*, and *Chlamydia* are factors in infertility, they may only be contributing factors rather than primary ones.

Because effective therapy may require treatment for several interrelated factors or organisms, it seems reasonable to first identify which organisms place patients at risk before attempting trial of antibiotics which may not be specific for these organisms. Our particular study was designed to determine the incidence of the genital mycoplasmas and chlamydia colonization in a group of fertile and infertile patients and to possibly identify a clinical subset of patients at a higher risk of colonization.

### SUBJECTS AND METHODS

This study consisted of a group of 80 private practice, infertile couples and a control group of 80 consecutive private practice couples with proven fertility in their first trimester of pregnancy. Information obtained for the investigation included:

- duration of infertility (at least 1 year);
- significant past medical history and social history;
- age and race of each partner;
- oral contraceptive (OC) or intrauterine device (IUD) use;
- history of pelvic inflammatory disease (PID) or sexually transmitted disease (STD);
- evidence of ovulation;
- hysterosalpingogram (HSG);
- laparoscopic findings;
- semen analysis and other male factors;
- results of cultures for *C. trachomatis*, *Mycoplasma*, *Ureaplasma*, *Neisseria gonorrhoeae*, aerobes, and anaerobes from the endocervix, endometrium, and peritoneal fluid obtained at time of laparoscopy, as previously described.[1–2]

Information taken from the control (fertile) group included:

- age and race of each partner;
- OC or IUD use;
- history of STD or PID;
- history of previous infertility work-up.

Similar cultures were collected from the endocervix for the previously mentioned organisms at time of initial examination for this group. Endometrial cultures utilized biopsy specimens obtained with Novak curette divided into 2 portions, 1 for routine histological studies and another for culture. At time of laparoscopy, a collection of peritoneal fluid was obtained. If fluid in the cavity was inadequate, lavage with normal saline and aspiration were performed. The fluid was centrifuged to pellet cellular material and then transferred to tissue culture. Direct specimens using swabs of the right oviduct were obtained in 16 patients. Specimens collected for *M. hominis* and *U. urealyticum* were done in a similar fashion and placed in a Mycotrans Mycoplasma Transport System (Irvine Scientific, Irvine, CA). These were refrigerated for 25 h, inoculated into a biphasic Mycotrim (Irvine Scientific) GU system, incubated, and then interpreted. *N. gonorrhoeae*, aerobic, and anaerobic cultures were obtained and evaluated by routine bacteriological techniques.[4] Chlamydia specimens were processed by tissue culture according to standard methodology.[5] For statistical analysis, Fisher's exact test or chi-square analysis was utilized. $P \leqslant 0.05$ was determined to be significant.

### RESULTS

The infertile and control groups were comparable with respect to the demographic characteristics listed in Table 1 ($P > 0.05$). None of the patients in either group related a history of PID and none of the patients in the fertile group related a history of infertility evaluation. The demographic data and laparoscopic results are listed in Table 2. At the time of laparoscopy, endometriosis was found in 33 (41%) of the patients. Adhesions were identified in 47 (70%) of all patients, but were present in only 31 (39%) of the patients without any evidence of endometriosis. Tubal occlusion, defined as distal occlusion as evidenced by hydrosalpinx, phimosis,

**TABLE 1. Demographic data of infertile and pregnant control groups**

| Population | Infertile group | Control group |
|---|---|---|
| No. | 80 | 80 |
| Age | | |
| Mean (years) | 30.9 | 29.1* |
| Range (years) | 20–40 | 18–39 |
| Race | | |
| Caucasian | 63 | 55 |
| Black | 8 | 12 |
| Hispanic | 5 | 9 |
| Others | 4 | 4 |
| History of PID | 0 | 0 |
| Duration of unexplained primary infertility (months) | 36.3 | — |
| Primary infertility | 43 (54%) | — |
| Secondary infertility | 37 (46%) | — |

*$P > 0.05$.

**TABLE 2. Demographic data and the laparoscopic findings according to ovulation status epidemiology (N = 67)**

| | Ovulation positive [N = 53 (79%)] | Ovulation negative [N = 14 (21%)] |
|---|---|---|
| Age | | |
| Mean (years) | 31.8 | 20.2 |
| Range (years) | 21–40 | 25–35 |
| Race | | |
| Caucasian | 41 | 14 |
| Hispanic | 3 | 0 |
| Black | 5 | 0 |
| Others | 4 | 0 |
| Infertility | | |
| Primary | 22 | 10 |
| Secondary | 31 | 4 |
| Average UPI[a] (months) | 38.2 | 28.2 |
| Miscellaneous | | |
| Term infants | 16 | 0 |
| Preterm infants | 2 | 0 |
| Miscarriage/abortions | 18 | 0 |
| Live child | 16 | 3 |
| Prior OC users | 36 | 7 |
| Prior OC non-users | 17 | 4 |
| Prior IUD users | 7 | 0 |
| Dysmenorrhea | 33 | 8 |
| Amenorrhea | 2 | 1 |
| Laparoscopic findings | | |
| Endometriosis | 25 (47%) | 8 (57%) |
| Pelvic adhesions | 40 (75%) | 7 (50%) |
| Tubal occlusion | 13 (25%) | 4 (29%) |
| Post-salpingectomy | 5 (9%) | 0 (0%) |
| Polycystic ovary disease | 3 (6%) | 2 (15%) |
| Fibroids | 5 (9%) | 1 (7%) |
| Hydrosalpinx | 1 (2%) | 1 (7%) |
| Dysfunction uterine bleeding | 0 | 1 (7%) |
| Cervical stenosis | 0 | 1 (7%) |
| Endopolyps | 0 | 1 (7%) |

[a]Unexplained primary infertility.

or severe adhesions occurring in at least 1 tube, was noted in 17 (21%) of the patients.

Cultures were obtained properly from 79 of the patients in the infertile group (Table 3). These included positive cervical cultures in 5 (6%) of the patients for *M. hominis* and 22 (28%) patients for *U. urealyticum*. Of the 76 endometrial cultures obtained, 2 (3%) had *M. hominis* and 4 (5%) grew *U. urealyticum*. Two of these 4 patients were found to have evidence of pelvic adhesions and histologic evidence of endometritis. Thirteen endometrial cultures were positive for other bacteria, with 1 patient also having a concomitant associated positive *Ureaplasma* culture. Bacteria isolated from the endocervix and endometrium consisted of aerobic and anaerobic species consistent with normal vaginal flora.

Of the 70 specimens of peritoneal fluid recovered, 1 (1%) grew *Ureaplasma*. This patient, who had had a previous unilateral salpingectomy for an ectopic pregnancy, was found to have extensive pelvic adhesions. Of the 20 tubal cultures obtained, all were negative. There were no positive cultures for *C. trachomatis* from any specimen, nor were there positive cultures for *N. gonorrhoeae*.

Comparing cervical cultures in the overall study group (Table 4) in patients with or without evidence of tubal occlusion as defined earlier, we found no statistical difference in the incidence of either of the 3 organisms found. In addition, when patients with tubal factor and unexplained infertility were combined, no statistical differences in their culture results were noted.

Thirteen of 17 patients with occlusion had histories of previous pelvic surgery. Of the 4 patients who had had no previous pelvic surgery, only 1 had a positive culture (*Ureaplasma*). In the subpopulation of 53 ovulating patients (i.e., a group of patients in which anovulation cannot be considered to be a contributing factor to the etiology of their infertility), the demographic characteristics were similar to the control group and infertile groups. Patients in the ovulatory group with laparoscopic

TABLE 3. Culture results in the infertile group

| Positive cultures | Ovulation positive | Ovulation negative | Uncertain or inconsistent intermediate ovulation |
|---|---|---|---|
| Cervix (n = 79)[a] | 52 | 14 | 13 |
| Bacteria[b] | 52 | 14 | 13 |
| GC[c] | 0 | 0 | 0 |
| *Chlamydia* | 0 | 0 | 0 |
| *Mycoplasma* | 1 | 1 | 3 |
| *Ureaplasma* | 14 | 2 | 6 |
| Endometrium (n = 76) | 51 | 13 | 12 |
| Bacteria | 10 | 1 | 2 |
| GC | 0 | 0 | 0 |
| *Chlamydia* | 0 | 0 | 0 |
| *Mycoplasma* | 0 | 0 | 2 |
| *Ureaplasma* | 0 | 1 | 0 |
| Peritoneal fluid (n = 70) | 48 | 11 | 11 |
| Bacteria | 0 | 0 | 0 |
| GC | 0 | 0 | 0 |
| *Chlamydia* | 0 | 0 | 0 |
| *Mycoplasma* | 0 | 0 | 0 |
| *Ureaplasma* | 0 | 1 | 0 |
| Fallopian tube (n = 20) | 16 | 4 | 0 |
| Bacteria | 0 | 0 | 0 |
| GC | 0 | 0 | 0 |
| *Chlamydia* | 0 | 0 | 0 |
| *Mycoplasma* | 0 | 0 | 0 |
| *Ureaplasma* | 0 | 0 | 0 |

[a]n = number of patients in group.
[b]Aerobic or anaerobic bacteria.
[c]GC = *N. gonorrhoeae*.

evidence of endometriosis were compared to those with pelvic adhesions (without endometriosis). The findings did not demonstrate a statistical difference in cervical culture results for *M. hominis* or *C. trachomatis*, but a statistically higher incidence of ureaplasma colonization ($P = 0.04$) was found in patients with endometriosis when compared to those with pelvic adhesions (36% vs. 12%). Sixteen of 25 patients with documented endometriosis in the ovulatory group also had concomitant adhesions. Furthermore, 18 of 23 patients with pelvic adhesions, but without endometriosis, had histories of previous pelvic surgery. Of the 5 patients in the pelvic adhesion group of ovulating patients without previous surgery, all had positive cervical cultures for *Mycoplasma* or *Ureaplasma*.

The outcome of pregnancy in the 80 patients in the fertile group was recorded. Sixty-seven (84%) of the patients carried their infants to term; 8 (10%) of the patients delivered prematurely; 4 (5%) of the patients had spontaneous abortions; and 1 of the patients had an ecotpic pregnancy. Cervical culture results in these patients revealed no statistical difference in colonization in any of these groups (Table 5).

### DISCUSSION

Female genital tract infections have long been implicated as a major cause of infertility. Gnarpe and Friberg[1] first suggested an etiologic role of *Mycoplasma* in infertility when they demonstrated a high frequency of positive cultures recovered from the cervices of women with unexplained infertility compared with those of the fertile pregnant control subjects. The unexplained infertility groups had normal basal body temperature charts, HSGs, and semen analyses. Others, however, have found no statistical difference in the incidence of *Mycoplasma* or *Ureaplasma* between fertile and infertile couples.[6,7] deLouvois et al.[7] studied 120 patients with infertility of various etiologies and found a 52% incidence of cervical ureaplasma. They also found a 55% incidence in 92 pregnant patients. Matthews et al.[8] and Nagata et al.[9] found similar results. Gump et al.[10] studied 20 patients with infertility for longer than 1 year and obtained cultures from the cervix and endometrium for *Mycoplasma* and *Ureaplasma*. They found no difference in the incidence of colonization with these organisms in patients with laparoscopic evidence of previous PID. They also found genital mycoplasma colonization in only 10 of 203 endometrial biopsy (EMB) specimens, and 1 of these was associated with endometrial inflammation.

During the same time, several investigators have attempted to demonstrate that treatment of these organisms would result in an increased pregnancy rate.[2,11] Most regimens involve attempted eradication of the organisms by treatment of both partners with either doxycycline or tetracycline, usually involving a 28-day course.[12,13]

The role of *C. trachomatis* in infertility has long been suggested.[14–18] Several investigators have demonstrated an association between serum antichlamydial antibodies and infertility in patients with tubal factors as the source of their infertility.[14–16] Despite these implications, few studies have been

**TABLE 4. Cervical culture results for diagnostic group**

| | No. patients | *Mycoplasma* (%) | *Ureaplasma* (%) | *Chlamydia* (%) |
|---|---|---|---|---|
| Control group (fertile) | 80 | 11 (14%)* | 23 (29) | 3 (4) |
| Infertile group | 79 | 4 (5) | 22 (28) | 0 |
| Ovulation positive | 53 | 1 (2) | 14 (26) | 0 |
| Pelvic adhesions without endometriosis | 25 | 1 (3) | 3 (12)** | 0 |
| Endometriosis | 25 | 0 (0) | 9 (36) | 0 |
| Tubal occlusion | 17 | 2 (12) | 4 (24) | 0 |
| At least one patent tube | 63 | 2 (3) | 17 (27) | 0 |

*The control group and the infertile groups were statistically different with respect to mycoplasma isolation ($P = 0.05$).
**The pelvic adhesion and the endometriosis groups were statistically different with respect to ureaplasma isolation ($P = 0.04$).

**TABLE 5. Cervical culture results in the control pregnant group (N = 80)**

| Outcome | No. | *Mycoplasma* positive (%) | *Ureaplasma* (%) | *Chlamydia* (%) |
|---|---|---|---|---|
| Term | 67 | 10 (15) | 19 (28) | 2 (3) |
| Preterm | 8 | 1 (13) | 3 (38) | 0 |
| Spontaneous abortion | 4 | 0 | 1 (25) | 1 (25) |
| Ectopic pregnancy | 1 | 0 | 0 | 0 |
| Total | 80 | 11 | 23 | 3 |

successful in isolating *C. trachomatis* from the endocervix or endometrium of infertile patients.

Cleary and Jones[19] studied 19 patients with positive serum anti-chlamydia antibodies greater than 1:32 and found *C. trachomatis* in 32% of their cervical cultures and 26% of their endometrial cultures. However, controls of seropositive fertile or seronegative infertile women were not done. In 1984, Kane et al.[20] found that 22% of 164 infertile women were seropositive for anti-chlamydial antibodies, while a control group had an 11% rate. However, they were unable to isolate *Chlamydia* from the cervices or fimbriae in any of the patients studied. However, in this investigation no statistical increase was found in the incidence of cervical colonization with *U. urealyticum* or *C. trachomatis* in the infertile groups. There was, however, a statistically significant increased incidence of *M. hominis* isolation in the pregnant group compared to the infertile group ($P = 0.05$). The lower incidence in the infertile group may have been the result of the previous use of antibiotics in earlier infertility work-ups or for other non-gynecologic indications. Additionally, seminal fluid cultures from the partners of our study group may have been helpful. Both Matthews et al.[8] and deLouvois et al.[7] found fewer than 5% of their couples studied had male positive cultures, with all female cultures being negative. Two studies in which couples were treated for *Ureaplasma* on the basis of positive semen cultures produced contradictory results. The first study by Rehewy et al.[21] failed to achieve a higher pregnancy rate, while Toth et al.[22] demonstrated that 60% of partners treated for *Ureaplasma* resulted in pregnancy compared to only 5% in cases where the post-therapy cultures remained positive. As stated earlier, confounding variables include the possibility of previous treatment of unrecognized infections.

This study did not document a higher incidence of active colonization with any of these 3 organisms in patients with infertility, tubal occlusion, or pelvic adhesions when compared to pregnant patients. However, one cannot exclude the possibility that these patients were infected in the past, at which time fertility damage might have occurred, and/or the possibility that subsequent treatment with antibiotics for any number of reasons resulted in a lower incidence of positive cultures. There does not appear to be a high rate of isolation of the genital mycoplasmas or *Chlamydia* at the time of infertility work-up. Either *C. trachomatis, M. hominis,* and *U. urealyticum* do not have a role in infertility or they are factors which manifest themselves prior to the formal work-up of the individual for infertility. Therefore, routine antibiotic treatment without culture confirmation at the time of infertility work-up in a similar patient population is not indicated. It may also be stated that in this, and perhaps similar infertility settings, culture of the endocervix for these organisms is unwarranted. However, work-ups performed by a physician at an earlier stage or in a population with a higher rate of PID may necessitate the consideration of obtaining cultures. If previous infections are proven at some point to be a factor in infertility, diagnosis and treatment of infection need to occur prior to the point at which patients seek infertility care. This challenge then falls on the patient's earlier obstetric and gynecologic care providers, perhaps even in the patient's teenage and early adulthood years before conception is often considered.

### REFERENCES

1. Gnarpe H, Friberg J: Mycoplasma and human reproductive failure. The occurrence of different mycoplasmas in couples with reproductive failure. Am J Obstet Gynecol 114:727, 1972.
2. Kundsin RB: Mycoplasma in genitourinary tract infections and reproductive failure. In Sturgis SH, Taymore ML (eds): Professions in Gynecology. Vol 5. New York: Grune & Stratton, p 275, 1970.
3. Friberg J, Gnarpe H: Mycoplasma and human reproductive failure. Pregnancies in "infertile" couples treated with doxycycline for *T-mycoplasmas*. Am J Obstet Gynecol 116: 23, 1973.
4. Berenson AB, Hamill HA, Martens MG, Faro S: Bacteriologic findings of post-cesarean endometritis in adolescents. Obstet Gynecol 75:627, 1990.
5. Ripa K, Mardh PA: Cultivation of *Chlamydia trachomatis* in cycloheximide treated McCoy cells. J Clin Microbiol 6:328, 1977.
6. Taylor-Robinson D: Evaluation of the role of *Ureaplasma urealyticum* in infertility. Pediatr Infect Dis 5:262, 1986.
7. deLouvis J, Blades M, Harrison RF, Hurley R, Stanley VC: Frequency of mycoplasma in fertile and infertile couples. Lancet 1:1073, 1974.
8. Matthews CD, Elmslie RG, Clapp KH, Svigos JM: The frequency of genital mycoplasma infection in human infertility. Fertil Steril 26:988, 1975.
9. Nagata Y, Iwaska T, Wada T: Mycoplasma infection and infertility. Fertil Steril 31:392, 1979.
10. Gump DE, Gibson M, Ashikaga T: Lack of association between genital mycoplasmas and infertility. N Engl J Med 310:937, 1984.
11. Horne HW, Hertig AT, Kundsin RB: Subclinical endometrial inflammation and *T-mycoplasmas:* A possible cause of human reproductive failure. Int J Fertil 18:266, 1973.
12. Harrison RF, Blades M, deLouvis J, Hurley R: Doxycycline treatment and human infertility. Lancet 1:605, 1975.
13. Hinton RA, Egdell LM, Andrews BE, Clark SK, Richmond SJ: A double-blind crossover study of the effect of doxycycline on mycoplasma infection and infertility. Br J Obstet Gynaecol 86:379, 1979.
14. Jones RB, Ardery BR, Hui SL, Cleary RE: Correlation between serum antibodies and tubal factor as a cause of infertility. Fertil Steril 38:553, 1982.
15. Moore DE, Foy HM, Daling JR, Grayston JT, Spadoni LR, Wang S, Kuo C, Eschenbach DA: Increased frequency of serum antibodies to *Chlamydia trachomatis* in infertility due to distal tubal disease. Lancet 2:574, 1982.
16. Sellors JW, Mohony JB, Chernesky MA, Rath DJ: Tubal factor infertility: An association with prior chlamydial infection and asymptomatic salpingitis. Fertil Steril 49: 451, 1988.
17. Anestad G, Lunde O, Moen M, Dalaker K: Infertility and chlamydial infection. Fertil Steril 48:787, 1987.
18. Henry-Suchet J, Utzmann C, De-Brux J, Ardoin P, Catalan F: Microbiological study of chronic inflammation associated with tubal factor infertility: Role of *Chlamydia trachomatis*. Fertil Steril 47:274, 1987.
19. Cleary RE, Jones RB: Recovery of *Chlamydia trachomatis* from the endometrium in infertile women with serum anti-chlamydial antibodies. Fertil Steril 44:233, 1985.
20. Kane JL, Woodland RM, Forsey T, Darougar S, Elder MG: Evidence of chlamydia infection in infertile women with and without fallopian tube obstruction. Fertil Steril 42:843, 1984.
21. Rehewy MSE, Thomas AJ, Hafez ESE, Brown WJ, Moghissi KS, Jaszczak S: *Ureaplasma urealyticum (T-mycoplasma)* in seminal plasma and spermatozoa from infertile and fertile volunteers. Eur J Obstet Gynecol Reprod Biol 8:247, 1978.
22. Toth A, Lesser ML, Brooks A, Labriola D: Subsequent pregnancies among 161 couples treated for *T-mycoplasma* genital-tract infection. N Engl J Med 308:505, 1983.

## References

[PDF_00489] Berenson A. B., Hammill H. A., Martens M. G., Faro S. (1990). Bacteriologic findings of post-cesarean endometritis in adolescents.. Obstet Gynecol.

[PDF_00534] Cleary R. E., Jones R. B. (1985). Recovery of Chlamydia trachomatis from the endometrium in infertile women with serum antichlamydial antibodies.. Fertil Steril.

[PDF_00485] Freberg J., Gnarpe H. (1973). Mycoplasma and human reproductive failure. 3. Pregnancies in "infertile" couples treated with doxycycline for T-0mycoplasmas.. Am J Obstet Gynecol.

[PDF_00477] Gnarpe H., Friberg J. (1972). Mycoplasma and human reproductive failure. I. The occurrence of different Mycoplasmas in couples with reproductive failure.. Am J Obstet Gynecol.

[PDF_00505] Gump D. W., Gibson M., Ashikaga T. (1984). Lack of association between genital mycoplasmas and infertility.. N Engl J Med.

[PDF_00511] Harrison R. F., de Louvois J., Blades M., Hurley R. (1975). Doxycycline treatment and human infertility.. Lancet.

[PDF_00530] Henry-Suchet J., Utzmann C., De Brux J., Ardoin P., Catalan F. (1987). Microbiologic study of chronic inflammation associated with tubal factor infertility: role of Chlamydia trachomatis.. Fertil Steril.

[PDF_00513] Hinton R. A., Egdell L. M., Andrews B. E., Clarke S. K., Richmond S. J. (1979). A double-blind cross-over study of the effect of doxycycline on mycoplasma infection and infertility.. Br J Obstet Gynaecol.

[PDF_00517] Jones R. B., Ardery B. R., Hui S. L., Cleary R. E. (1982). Correlation between serum antichlamydial antibodies and tubal factor as a cause of infertility.. Fertil Steril.

[PDF_00537] Kane J. L., Woodland R. M., Forsey T., Darougar S., Elder M. G. (1984). Evidence of chlamydial infection in infertile women with and without fallopian tube obstruction.. Fertil Steril.

[PDF_00500] Matthews C. D., Elmslie R. G., Clapp K. H., Svigos J. M. (1975). The frequency of genital mycoplasma infection in human fertility.. Fertil Steril.

[PDF_00520] Moore D. E., Spadoni L. R., Foy H. M., Wang S. P., Daling J. R., Kuo C. C., Grayston J. T., Eschenbach D. A. (1982). Increased frequency of serum antibodies to Chlamydia trachomatis in infertility due to distal tubal disease.. Lancet.

[PDF_00503] Nagata Y., Iwasaka T., Wada T. (1979). Mycoplasma infection and infertility.. Fertil Steril.

[PDF_00541] Rehewy M. S., Thomas A. J., Hafez E. S., Brown W. J., Moghissi K. S., Jaszczak S. (1978). Ureaplasma urealyticum (T-mycoplasma) in seminal plasma and spermatozoa from infertile and fertile volunteers.. Eur J Obstet Gynecol Reprod Biol.

[PDF_00492] Ripa K. T., Mårdh P. A. (1977). Cultivation of Chlamydia trachomatis in cycloheximide-treated mccoy cells.. J Clin Microbiol.

[PDF_00524] Sellors J. W., Mahony J. B., Chernesky M. A., Rath D. J. (1988). Tubal factor infertility: an association with prior chlamydial infection and asymptomatic salpingitis.. Fertil Steril.

[PDF_00546] Toth A., Lesser M. L., Brooks C., Labriola D. (1983). Subsequent pregnancies among 161 couples treated for T-mycoplasma genital-tract infection.. N Engl J Med.

